# A Novel Role for p115RhoGEF in Regulation of Epithelial Plasticity

**DOI:** 10.1371/journal.pone.0085409

**Published:** 2014-01-23

**Authors:** Swapnil S. Kher, Amanda P. Struckhoff, Arthur S. Alberts, Rebecca A Worthylake

**Affiliations:** 1 Department of Pharmacology and Experimental Therapeutics, LSU Health Sciences Center, New Orleans, Louisiana, United States of America; 2 Stanley S. Scott Cancer Center, LSU Health Sciences Center, New Orleans, Louisiana, United States of America; 3 Center for Cancer and Cell Biology, Van Andel Research Institute, Grand Rapids, Michigan, United States of America; 4 Department of Oral Biology, LSU Health Sciences Center, New Orleans, Louisiana, United States of America; NCMLS, Radboud University Nijmegen Medical Center, The Netherlands

## Abstract

Epithelial plasticity plays a critical role during physiological processes, such as wound healing and tissue regeneration, and dysregulation of epithelial plasticity can lead to pathological conditions, such as cancer. Cell-cell junctions are a critical feature of epithelial cells and loss of junctions is associated with acquisition of mesenchymal features, such as enhanced protrusion and migration. Although Rho has been implicated in regulation of junctions in epithelial cells, the role of Rho signaling in the regulation of epithelial plasticity has not been understood. We show that members of the RGS RhoGEFs family play a critical role in regulation of epithelial cell-cell junctions in breast epithelial cells. We identify a novel role for p115RhoGEF in regulation of epithelial plasticity. Loss of p115RhoGEF leads to decreased junctional E-cadherin and enhanced protrusiveness and migration. Conversely, overexpression of p115RhoGEF enhanced junctional E-cadherin and inhibited cell protrusion and migration. siRNA screen of 23 Rho effectors showed that members of the Diaphanous-Related Formin (DRF) family are required for p115RhoGEF-mediated changes in epithelial plasticity. Thus, our data indicates a novel role for p115RhoGEF in regulation of epithelial plasticity, which is dependent on Rho-DRF signaling module.

## Introduction

Epithelial cells line the tissues of many organs and are highly differentiated to execute specific functions required by the breast, colon and lung. Cell-cell contacts defined by tight junctions, adherens junctions and desmosomes result in apical-basolateral polarity that is essential for proper epithelial cell function. These cells help maintain tissue homeostasis and are generally non-motile. Intriguingly, epithelial cells can also transiently lose their cell-cell junctions and other epithelial cell characteristics to become more mesenchymal with an elongated morphology and protrusive lamellipodia that support motility. This occurs in normal physiological processes such as tubulogenesis and branching in the mammary gland, or tissue reorganization during wound healing. However, this inherent plasticity in the display of an epithelial phenotype also enables pathophysiological consequences during diseases such as organ fibrosis or tumor metastasis [1].

Adherens junctions are formed by E-cadherin complexes that physically link neighboring epithelial cells, and are a defining feature of epithelial cells. Thus, detailed knowledge of the signaling pathways that control them is important for understanding epithelial cell plasticity. RhoA is a small GTPase that regulates cell-cell junctions, however its precise role is complex. Some studies show that too much RhoA disrupts cell-cell junctions, while others show that RhoA is required for these same structures [2], [3], [4]. Similarly, RhoA plays a complex role in the regulation of actin structures associated with a motile mesenchymal phenotype. High levels of RhoA can block actin-rich protrusions, yet it can also be required for protrusion and motility [5], [6], [7]. Sometimes these disparate findings are explained by cell type specific differences, but the molecular mechanisms responsible have not been identified. More recent investigations into the details of RhoA signaling suggest that nuanced control of its activity and coupling to selective downstream effectors are important determinants of context dependent RhoA signaling outcomes [8], [9].

Rho GTPases are activated by GEFs (guanine nucleotide exchange factors), of which there are 69 members in the Dbl family of RhoGEFs. The large number of potential activators suggest that individual RhoGEFs may determine selective RhoA activation and signaling pathways, which could mechanistically explain the diversity of RhoA signaling outcomes [10]. In our study, we used siRNA to knockdown the 3 members of a subfamily of RhoGEFs containing an RGS (regulator of G-protein signaling) domain to determine the effect on adherens junctions in breast tumor epithelial cells. These studies showed that p115RhoGEF was selectively required for intact E-cadherin structures at cell-cell junctions. p115RhoGEF has previously been studied in smooth muscle cells, neutrophils and leukocytes [11], [12], [13], but not in epithelial cells; thus our findings that p115RhoGEF promotes adherens junctions and inhibits breast tumor epithelial cell motility is novel. Furthermore, a rigorous gene expression study in epithelial cells undergoing transition to a mesenchymal phenotype showed that p115RhoGEF expression was downregulated 4 fold when the epithelial phenotype was lost [14]. Together with our new findings, this indicates that p115RhoGEF is a significant regulator of epithelial cell plasticity.

## Results

### RGS GEF knockdown show distinct effects on adherens junctions

**Figure 1:**
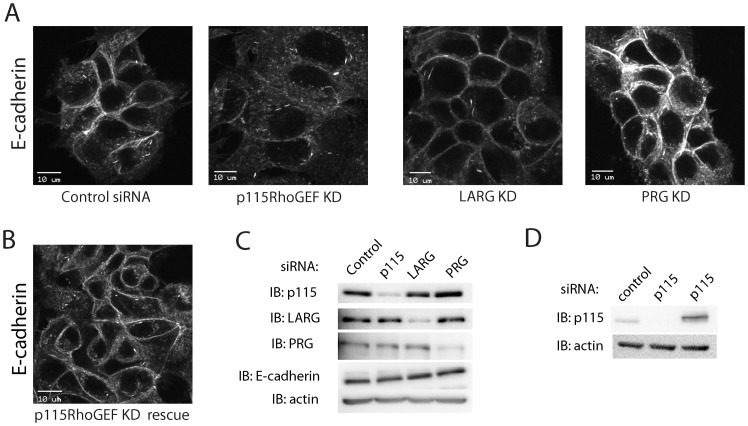
RGSGEF knockdown show distinct effects on adherens junctions. A) MCF7 cells were transfected with siRNA to target either p115RhoGEF, LARG, or PRG RGS RhoGEF. Immunofluoresence staining for E-cadherin showed a distinct effect on junctional morphology. Knockdown of p115RhoGEF (p115RhoGEF KD) disrupted adherens junctions, knockdown of PRG (PRG KD) promoted junctions, whereas knockdown of LARG (LARG KD) did not show any effect as compared to cells treated with non–targeting control siRNA. B) siRNA-resistant p115RhoGEF was stably expressed in MCF7 cells. Expression of siRNA resistant p115RhoGEF (p115RhoGEF KD rescue) in cells where endogenous p115RhoGEF had been knocked down restored junctional morphology. C) Immunoblot demonstrating siRNA knockdown of each of the RGS RhoGEFs does not affect expression of other members of the family. Immunoblot for E-cadherin showed that the total level of cellular E-cadherin remained the same in each of the three knockdowns. D) Representative immunoblot demonstrating expression of siRNA resistant p115RhoGEF in cells that were treated with p115RhoGEF siRNA. Scale bars represent 10 µm.

Although RhoGTPases have been implicated in regulation of cell-cell junctions, the complex role played by the Rho subfamily in junctional regulation has not been clearly understood. To understand the fine-tuning of Rho activity at cell-cell junctions, we used siRNA to knockdown the three members of RGS family of GEFs comprising p115 RhoGEF, LARG and PRG in MCF7 cells. Each siRNA selectively reduced gene expression of its target gene without altering the expression of other RGS RhoGEF family members (Figure 1C). We found that knockdown of each of the three GEFs had a distinct effect on junctional morphology. Immunofluorescent detection of E-cadherin localization showed that knockdown of p115RhoGEF disrupted localization of E-cadherin at adherens junctions, whereas PRG knockdown showed enhanced junctions as has been recently published [15]. LARG knockdown did not have an identifiable effect on junctions (Figure 1A). Since p115 RhoGEF knockdown clearly disrupted adherens junctions we reasoned that p115RhoGEF is required for regulation of junctional integrity and decided to focus on p115RhoGEF to study its effects on adherens junction plasticity. Expression of a siRNA-resistant variant of p115RhoGEF in knockdown cells restored adherens junctions, confirming that the observed effect on adherens junctions was specific to loss of p115RhoGEF and was not an off-target effect of siRNA transfection (Figure 1B). Interestingly, we also found that even though knockdown of either p115RhoGEF or PRG had a dramatic effect on junctional morphology, immunoblot for E-cadherin in each of the three RGS GEF knockdowns showed no change in E-cadherin expression suggesting a redistribution of E-cadherin upon loss of p115RhoGEF and PRG (Figure 1C). Thus, our results from siRNA knockdown experiments indicate a novel role for RGS RhoGEFs in the regulation of epithelial adherens junctions.

### Loss of p115RhoGEF decreases junctional E-cadherin and promotes protrusions

**Figure 2:**
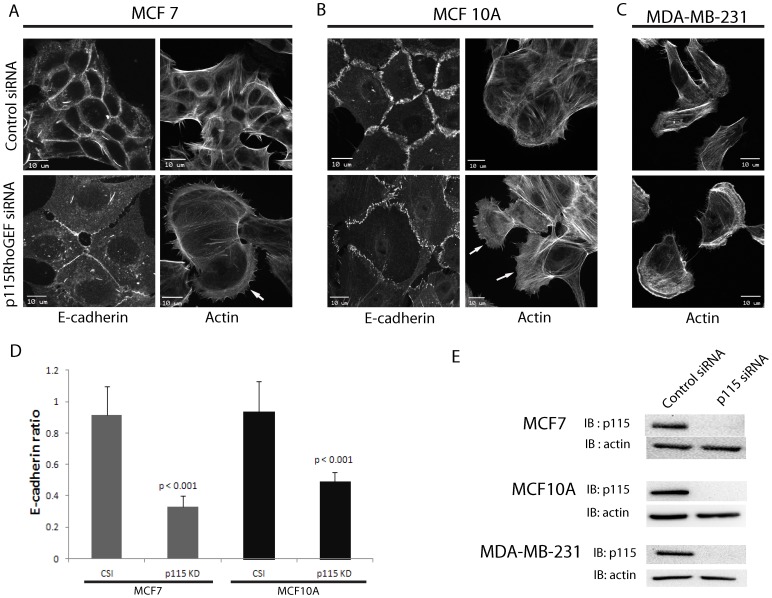
Loss of p115RhoGEF decreases junctional E-cadherin and promotes protrusions. A) p115RhoGEF was knocked down in MCF7 cells and stained for E-cadherin and Actin. E-cadherin staining showed decreased junctional E-cadherin as compared to cells treated with control siRNA. Actin staining using AlexaFluor594-labeled phalloidin showed enhanced protrusions as compared to control B) p115RhoGEF knockdown in MCF10A cells showed decreased junctional E-cadherin and enhanced protrusions as compared to control cells. C) Actin staining in p115RhoGEF-depleted MDA-MB-231 cells shows enhanced lamella as compared to cells treated with control siRNA. D) Quantitation of the ratio of junctional over cytoplasmic E-cadherin in MCF7 and MCF10A cells treated with p115RhoGEF siRNA showed a significant decrease (mean ± S.E.) in junctional E-cadherin as compared to cells treated with control siRNA. A total of 37 cell-cell junctions for MCF7 cells and 33 cell-cell junctions for MCF10A from 3 separate experiments were analyzed. p-value was calculated using 2 tailed students t-test E) Representative immunoblots showing knockdown of p115RhoGEF in each of the three cell types. Scale bars represent 10 µm.

Cellular transformation in epithelial carcinomas is characterized by acquisition of mesenchymal characteristics such as loss of cell-cell junctions and enhanced actin-rich protrusions [16]. We used siRNA to test if p115RhoGEF depletion would promote acquisition of these morphological characteristics. Depletion of p115RhoGEF in MCF7 cells resulted in a significant (3-fold) decrease in junctional E-cadherin localization as compared to cells treated with control siRNA (Figure 2A and 2D). To determine if loss of p115RhoGEF also lead to corresponding changes in the actin cytoskeleton and enhanced protrusions, we stained the cells for actin to look for changes in the actin cytoskeleton. p115RhoGEF depletion resulted in enhanced actin-rich protrusions at the cell periphery (Figure 2A). MCF7 cells are a breast carcinoma cell line, and although they retain many epithelial cell characteristics, they are transformed and may have acquired alterations in cytoskeletal signaling pathways. To confirm the above observations in non-transformed cells, we repeated our experiments in the MCF10A breast epithelial cell line and found that similar to our results in MCF7, loss of p115RhoGEF caused a significant (2-fold) decrease in junctional E-cadherin and enhanced actin-rich protrusions (Figure 2B and 2D). The results from both MCF7 and MCF10A cells show changes in E-cadherin and actin cytoskeleton upon loss of p115RhoGEF. To determine if loss of p115RhoGEF would further promote these features in cells that already display mesenchymal features, we also repeated the same experiment in MDA-MB-231 cells, which exhibit mesenchymal features such as actin-rich protrusions and absence of E-cadherin. Loss of p115RhoGEF showed enhanced actin-rich protrusions as compared to control (Figure 2C).

These results show that loss of p115RhoGEF in breast epithelial cells decrease epithelial features such as E-cadherin mediated adherens junctions and enhance mesenchymal features by promoting actin-rich protrusions. Also, results from experiments in MDA MB 231 indicate that the effect of p115RhoGEF on the actin cytoskeleton could be independent of its effect on cell-cell junctions.

### Overexpression of p115RhoGEF enhances junctional E-cadherin and blocks protrusions

**Figure 3:**
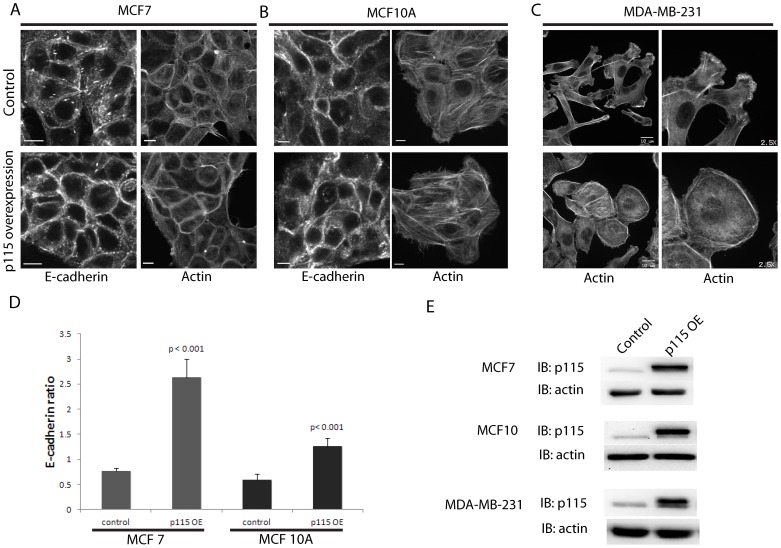
Overexpression of p115RhoGEF enhances junctional E-cadherin and blocks protrusions. A) MCF7 cells that overexpressed p115RhoGEF were stained for E-cadherin and actin. p115RhoGEF overexpressors showed an increase in junctional E-cadherin as well as increase in junctional actin as compared to control cells. B) Overexpression of p115RhoGEF in MCF10A cells showed enhanced junctional E-cadherin and a more subtle effect on junctional actin as compared to control cells that overexpressed GFP alone. C) Actin staining of MDA-MB-231 cells that overexpressed p115RhoGEF showed loss of protrusions and a change in morphology with a clear ring of actin at the cell periphery. D) Quantitation of the ratio of junctional over cytoplasmic E-cadherin in MCF7 and MCF10A cells showed a significant increase in junctional E-cadherin in cells that overexpressed p115RhoGEF (mean ± SE) as compared to control. A total of 45 cell-cell junctions for both MCF7 and MCF10A from 3 separate experiments were analyzed. p-value was calculated using 2 tailed t-test. E) Representative immunoblot showing overexpression of p115RhoGEF (p115 OE) as compared to endogenous levels in control cells in each of the three cell lines. Scale bars represent 10 µm.

Since p115RhoGEF depletion decreased junctional E-cadherin and enhanced actin-rich protrusions, we hypothesized that overexpression of p115RhoGEF would have the opposite effect and promote a more epithelial-like phenotype. We stably overexpressed p115RhoGEF using retroviral transduction in MCF7, MCF10A and MDA-MB-231 cells and confirmed overexpression by immunoblot (Figure 3E). Overexpression of p115RhoGEF in MCF7 cells significantly increased (4-fold increase) junctional E-cadherin localization and also enhanced junctional actin as compared to control MCF7 cells that expressed GFP alone (Figure 3A and 3D). The results were similar in MCF10A cells where we observed a significant (2-fold) increase in junctional E-cadherin and a subtle increase in junctional actin upon overexpression of p115RhoGEF (Figure 3B and 3D). Interestingly overexpression of p115RhoGEF in MDA-MB-231 cells completely blocked their actin-rich protrusions and showed an altered morphology with a prominent actin ring at the periphery (Figure 3C). Interestingly, loss or gain of p115RhoGEF did not change the localization of the tight junction marker ZO-1 in MCF7 cells (Figure S1). This suggests that the effect of p115RhoGEF on cell junctions is selective for cadherin-mediated adherens junctions. Thus, based on our data from three different cell lines, we conclude that p115RhoGEF promotes epithelial features such as adherens junctions and blocks mesenchymal features such as protrusions. Our results from the p115 RhoGEF depletion and overexpression experiments indicate a pivotal role for p115RhoGEF in the regulation of epithelial plasticity.

### Expression levels of p115RhoGEF affect migration

**Figure 4:**
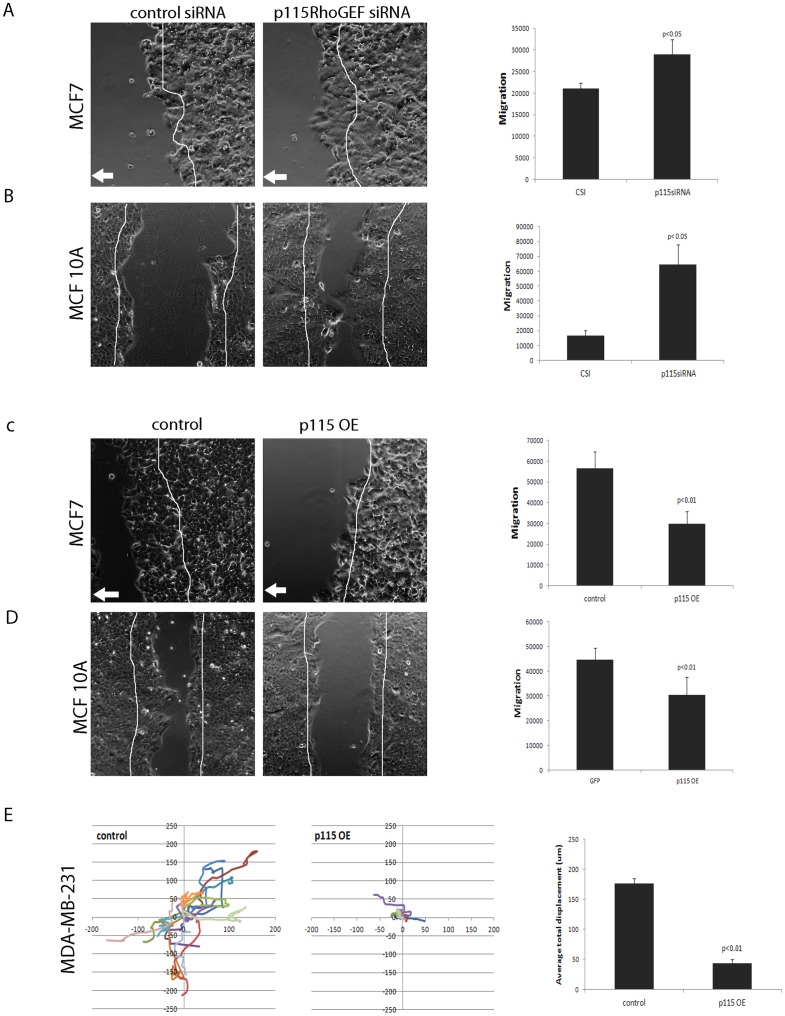
Expression levels of p115RhoGEF affect cell migration. A) Scratch wound assay showed a moderate but significant increase in migration of p115RhoGEF-depleted MCF7 cells as compared to cells treated with control siRNA. Phase contrast images show boundaries of wounds in cell monolayer at the end of migration assay (24 hrs) while the boundary of the wounds at the beginning of the assay (0 hrs) are indicated by superimposed white lines in order to reduce the number of images shown. For both control and p115RhoGEF-depleted cells, three separate locations were imaged at 0 h and 24 h after making the scratch and the difference in wound area was determined using ImageJ software. White arrow indicates direction of cell migration. Migration was measured by measuring the difference in area covered by the cell monolayer at 0 h and 24 h. Cells treated with p115RhoGEF siRNA showed a significant increase in migration as compared to control siRNA treated cells (n = 3). B) Scratch wound assay in MCF10A cells showed a significant increase in migration of p115RhoGEF-depleted MCF10A cells as compared to cells treated with control siRNA (n = 3). C) Scratch wound assay showed a significant decrease in migration of MCF7 cells that overexpressed p115RhoGEF as compared to control cells that expressed GFP alone. D) Scratch wound assay showed a modest but significant decrease in migration of MCF10A cells that overexpressed p115RhoGEF as compared to control cells. E) Results for MDA-MB-231 single cell migration assay. Control (GFP) and p115RhoGEF-overexpressing (p115 OE) MDA-MB-231 cells were plated on MatTek plates and images were collected every 30 min for 6 h. During image capture, cells were maintained at 37°C and 5% CO2 using a Live Cell™ chamber. Individual cell positions in sequential images were determined using the cell tracking tool in Slidebook software and x-y co-ordinates with starting points adjusted to (0,0) were plotted. Migration of 5 cells was measured in each experiment and data was pooled from three individual experiments. p115-OE MDA-MB-231 cells displayed decreased migration as compared to control cells that expressed GFP alone. Quantitation of average total displacement which is a measure of the total distance travelled by the migrating cell showed a significant decrease in cells that overexpressed p115RhoGEF as compared to control cells that expressed GFP alone.

Although epithelial cells are normally non-migratory, they can lose their epithelial characters and adopt a more migratory mesenchymal phenotype during normal physiological processes such as wound healing, or during pathophysiological processes, such as cellular transformation during tumor progression [16]. As we hypothesized that p115RhoGEF is a regulator of epithelial plasticity, we predicted that p115RhoGEF expression would be inversely related to cell migration. We used a scratch wound assay to determine the effect of p115RhoGEF depletion or overexpression on migration in MCF7 and MCF10A cells. Loss of p115RhoGEF in MCF7 showed a modest but significant 30% increase in basal cell migration. (Figure 4A). In MCF10A, p115RhoGEF knockdown showed a significant 3-fold increase in basal migration (Figure 4B). We did not test the effect of p115RhoGEF depletion in MDA-MB-231 cells since these cells are already highly migratory. Conversely, overexpression of p115RhoGEF in MCF7 cells significantly decreased (2-fold) basal cell migration (Figure 4C). A similar effect was seen in MCF10A cells where overexpression of p115RhoGEF decreased (1.5-fold) basal migration as compared to control cells that expressed GFP alone (Figure 4D). Since MDA-MB-231 cells do not form a monolayer, we used single cell migration assay to measure changes in MDA-MB-231 cell migration. We observed that overexpression of p115RhoGEF almost completely blocked migration in MDA-MB-231 cells that stably cells overexpressed p115RhoGEF with a 4-fold decrease in average total displacement as compared to control cells that expressed GFP alone (Figure 4E). These results confirm that expression levels of p115RhoGEF have an inverse effect on cell migration in breast epithelial and breast carcinoma cells.

### p115RhoGEF activates RhoA and the catalytic activity of p115RhoGEF is required for its regulation of epithelial plasticity

**Figure 5:**
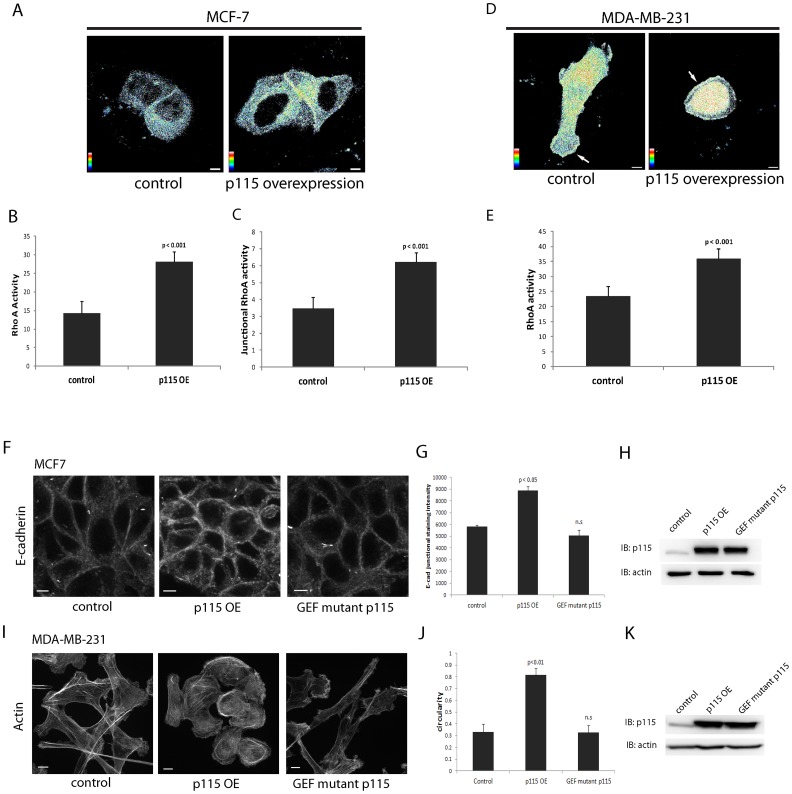
Overexpression of p115RhoGEF leads to activation of RhoA and catalytic activity of p115RhoGEF is required for its regulation of epithelial plasticity. A) Control and p115-OE MCF7 cells and cells were transfected with a FRET-based RhoA biosensor. Cells were imaged for CFP and FRET and FRET/CFP ratio representative of RhoA activity was calculated. Warmer colors correspond to high RhoA activity. High RhoA activity was seen at cell-cell junctions and within the cell body in cells that overexpressed p115RhoGEF as compared to control MCF7 cells. B) Total RhoA activity was quantitated by calculating FRET intensity per unit area of the cell from a total of 30 cells (n = 3). Cells that overexpressed p115RhoGEF showed a significant increase in total RhoA activity as compared to control cells (mean ± SE). C) Junctional RhoA activity was quantitated by calculating FRET intensity per unit length of the junction from a total of 30 cells (n = 3). Cells that overexpressed p115RhoGEF showed a significant increase in junctional RhoA as compared to control cells (mean ± SE). D) Control and p115-OE MDA-MB-231 cells were transfected with the FRET-based RhoA biosensor. High RhoA activity was seen around the cell periphery and within the entire cell body of p115-OE MDA-MB-231 cells as compared to control cells that showed RhoA activation at protrusions and in areas in the rear of the cell. E) Total RhoA activity was quantitated by calculating FRET intensity per unit area of the cell from 25 cells (n = 3). p115-OE MDA-MB-231 cells showed a significant increase in total RhoA activity as compared to control cells (mean ± SE). F) MCF7 cells overexpressing a catalytically inactive p115RhoGEF^DH mutant^ (GEF mutant p115) showed no change in junctional E-cadherin staining intensity as compared to cells that overexpressed wild type p115RhoGEF G) Quantitation of junctional staining intensity of E-cadherin in cells that overexpressed p115RhoGEF^DH mutant^ did not show a significant difference as compared to control cells (average ± S.E.). 150 cell-cell junctions were analyzed from 3 separate experiments. H) Representative immunoblots showing expression of p115RhoGEF^DH mutant^ in MCF7. I) Actin staining of p115RhoGEF^DH mutant^–expressing MDA-MB -231 cells did not show the change in morphology seen upon overexpression of wild type p115RhoGEF. J) Quantitation of change in morphology in terms of circularity of the cells reveal no significant difference between control cells and cells that overexpressed p115RhoGEF^DH mutant^ (mean ± SE). 75 cells were analyzed from 3 separate experiments. K) Representative immunoblots showing overexpression of p115RhoGEF^DH mutant^ as compared to endogenous p115RhoGEF in control cells.

While some RhoGEFs are capable of activating multiple small GTPases, the catalytic activity of p115RhoGEF has been thoroughly studied biochemically and has been shown to selectively activate Rho (RhoA, B, or C), but not Rac, Cdc42, or TC10 [17], [18]. Since overexpression of p115RhoGEF showed a clear phenotype in MCF7 and MDA-MB-231 cells, we used these cells to confirm that overexpression of p115RhoGEF resulted in changes in RhoA activity. We used a well-characterized Fluorescent Resonance Energy Transfer (FRET) biosensor that can detect active RhoA in live cells [15], [19], [20]. Measurement of total RhoA activity using this FRET-based RhoA biosensor revealed significant enhancement in RhoA activation in both MCF7 (Figure 5A & 5B) and MDA-MB-231 (Figure 5D & 5E) cells upon overexpression of p115RhoGEF. Overexpression of p115RhoGEF in MCF7 also showed significantly enhanced RhoA activity at cell-cell junctions as compared to control cells (Figure 5A & 5C). In the case of MDA-MB-231 cells, overexpression of p115RhoGEF showed strong RhoA activity at cell periphery and the entire internal cell body. This is in contrast to control cells, where RhoA activation was restricted to cell protrusions and to the rear of the cell (Figure 5D). Rho has been reported to regulate epithelial morphology by regulating cell-cell junctions and E-cadherin distribution [21], [22]. Since overexpression of p115RhoGEF showed enhanced junctional E-cadherin and blocked protrusions, we wanted to test if the GEF catalytic activity of p115RhoGEF was necessary for this effect. We made a point mutation in the DH domain of p115RhoGEF to make a catalytically inactive mutant of p115RhoGEF (p115RhoGEF^DHmutant^). Analogous mutation of the tyrosine residue within the conserved QRITKY sequence in Dbl homology domain of other RhoGEFs, such as p114RhoGEF, GEF-H1and Lbc, have been previously shown to inactivate their GEF exchange activities [23], [24], [25]. Stable overexpression of p115RhoGEF^DHmutant^ in MCF7 cells did not enhance junctional E-cadherin localization, which was seen upon overexpression of the wild-type p115RhoGEF (Figure 5F and Figure 3). The increase in junctional E-cadherin that was observed upon overexpression of wild type p115RhoGEF dropped to control levels upon overexpression of p115RhoGEF^DHmutant^ (Figure 5G). Similarly, overexpression of p115RhoGEF^DHmutant^ in MDA-MB-231 showed that the catalytic activity of p115RhoGEF was required for the effect of ectopic p115RhoGEF overexpression on protrusions (Figure 5I). Quantitation of change in morphology in terms of circularity of cells showed no significant change upon expression of p115RhoGEF^DHmutant^ as compared to control cells that overexpressed GFP alone (Figure 5J). Our results show that overexpression of p115RhoGEF leads to activation of RhoA, and since the effects of overexpression on junctional E-cadherin and protrusions are blocked upon inactivation of the GEF catalytic activity of p115RhoGEF, these results suggest that the overexpression phenotype of p115RhoGEF is dependent upon its GEF catalytic activity.

### Members of the Diaphanous Related Formin (DRF) family are required for p115RhoGEF regulation of epithelial plasticity

**Figure 6:**
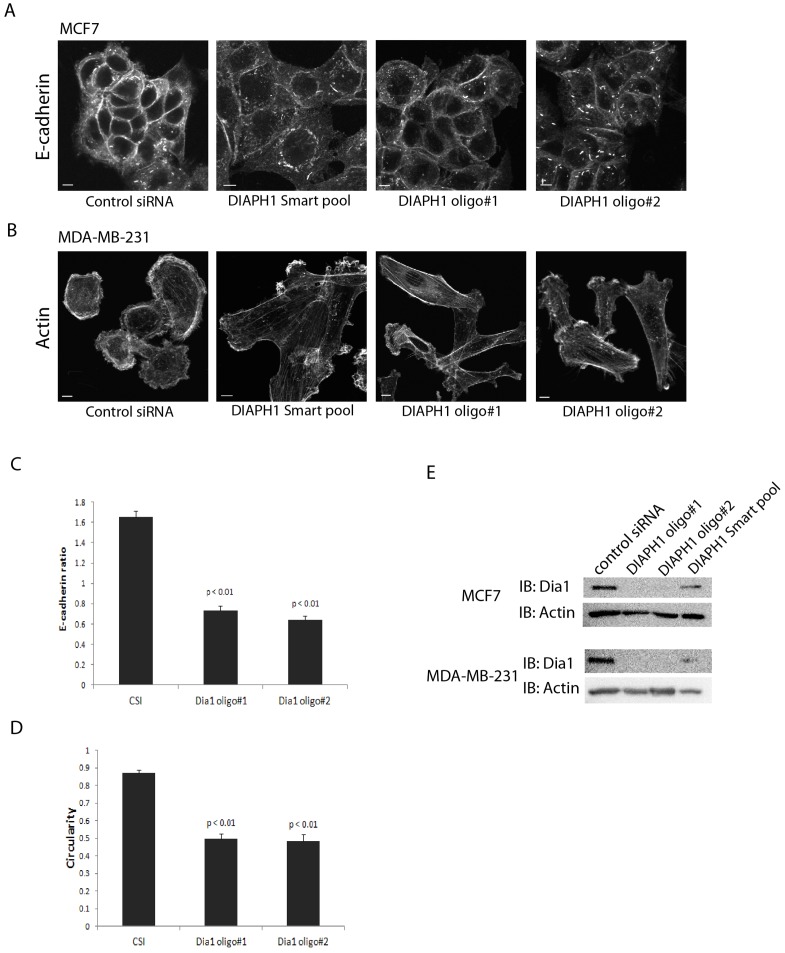
Members of diaphanous related formin (DRF) family are required to mediate regulation of epithelial plasticity by p115RhoGEF. A) p115-OE MCF7 cells were transfected with SMARTpool™ siRNA or individual siRNA oligos targeting *DIAPH1*. *DIAPH1*-depleted p115-OE MCF7 cells showed decreased junctional E-cadherin as compared to *DIAPH1-*expressing p115-OE MCF7 cells. B) p115-OE MDA-MB-231 cells were treated with SMARTpool™ siRNA or individual siRNA oligos targeting *DIAPH1*. *DIAPH1* knockdown blocked the change in morphology seen in cells that overexpressed p115RhoGEF. C) Quantitation of E-cadherin ratio in p115-OE MCF7 cells showed a significant decrease in upon *DIAPH1* knockdown as compared to cells that were treated with control siRNA (mean ± SE). 90 cell-cell junctions were analyzed from 3 experiments. D) Quantitation of morphology in terms of circularity in p115-OE MDA-MB-231 cells showed a significant change upon *DIAPH1* knockdown as compared to cells that were treated with control siRNA (mean ± SE). 75 cells were analyzed from 3 experiments E) Representative immunoblots showing knockdown of *DIAPH1* upon treatment with *DIAPH1* oligo#1, *DIAPH1* oligo#2 and *DIAPH1* SMARTpool™ siRNA in MCF7 or MDA-MB-231 cells that overexpressed p115RhoGEF. Scale bars represent 10 µm.

Multiple Rho effectors have been reported to regulate adherens junctions and actin cytoskeleton in epithelial cells [21], [22], [26], [27]. To identify the Rho effector(s) that mediate p115RhoGEF dependent regulation of epithelial plasticity, we used our p115RhoGEF overexpression (p115RhoGEF-OE) system which shows the p115RhoGEF-dependent promotion of adherens junctions and inhibition of protrusions. We performed a siRNA screen targeting 23 known RhoA effectors in p115RhoGEF-OE MCF7 and MDA-MB-231 cells, since both the cell types had displayed a robust p115RhoGEF-OE phenotype in earlier experiments. Of the 23 RhoA effectors that were targeted in the screen, we found that only knockdown of effectors belonging to the Diaphanous Related Formin (DRF) family of proteins i.e. *DIAPH1*, *DIAPH2*, *DIAPH3* and *DAAM* were able to block enhanced junctional E-cadherin in MCF7 cells and change in morphology of MDA-MB-231 due to p115RhoGEF expression. Our observation that knockdown of the DRF family of Rho effectors blocked the p115RhoGEF-overexpression phenotype in two different cell types strongly suggested the involvement of the DRF effectors in p115RhoGEF-mediated promotion of an epithelial phenotype. Since DIAPH1 is the more well-studied member of the DRF family, we decided to focus on DIAPH1 as a representative candidate of this family. To confirm that the reversal of the p115-OE phenotype was specific for *DIAPH1* knockdown, we also used a second independent set of siRNA oligos to deplete *DIAPH1* in these experiments. Depleting *DIAPH1* using either SMARTpool™ or individual oligos in MCF7 p115RhoGEF-OE cells produced a significant decrease in junctional E-cadherin localization, as compared to MCF7 p115RhoGEF-OE cells that expressed *DIAPH1* (Figure 6A and 6C). Similarly in MDA-MB-231 cells, knockdown of *DIAPH1* using either SMARTpool™ or individual siRNA oligos significantly blocked the change in morphology previously associated with overexpression of p115RhoGEF (Figure 6B and 6D). Thus, our results demonstrate that DRF family members are required for p115RhoGEF-dependent adherens junction enhancement and inhibition of actin-based protrusions in MCF7 and MDA-MB-231 cells.

## Discussion

Epithelial plasticity plays a central role in a number of physiological processes such as morphogenesis, maintenance of tissue homeostasis, wound healing, and tissue regeneration. Loss of regulation of epithelial plasticity results in pathological conditions such as fibrosis and tumor metastasis [1]. During these processes, cells lose their characteristic epithelial features such as cuboidal shape and strong cell-cell junctions and acquire a more mesenchymal morphology characterized by enhanced protrusions and migration. Rho and its downstream signaling effectors have been implicated in regulation of plasticity by modulation of the epithelial characteristics listed above. However, the reports on the role of Rho signaling in the maintenance of the epithelial phenotype are somewhat contradictory, and contribution of Rho-activated signaling pathways in regulating epithelial plasticity is not clearly understood [21], [28]. RhoGEFs (Guanine nucleotide Exchange Factors) and RhoGAPs (GTPase Activating Proteins) have emerged as excellent candidates to explain the multifaceted regulation of Rho signaling. While RhoGEFs are best understood for their direct activation of Rho GTPases, they can also influence downstream signaling from Rho effectors, restrict the spatial activation of Rho GTPases, create scaffolding platforms to recruit specific Rho effectors to produce differential signaling outputs and can also couple with a specific RhoA effector to form a signaling module to regulate a specific biological function [8], [9], [29], [30], [31], [32]. siRNA knockdown of the three RGS RhoGEF family members revealed a novel role for these RhoGEFs in the regulation of cell-cell junctions. Our study found that the expression level of p115RhoGEF determined the balance between adherens junctions and membrane protrusions to regulate epithelial plasticity. Thus, while p115RhoGEF has been demonstrated to regulate aspects of endothelial and immune cell biology, our data reveal a novel role for p115RhoGEF in the regulation of epithelial plasticity.

We used both knockdown and overexpression approaches to investigate the relationship between p115RhoGEF expression levels and promotion of an epithelial-like phenotype in both non-transformed and transformed breast cell lines. Loss of p115RhoGEF in MCF7 cells resulted in the loss of epithelial features, with an observed decrease in junctional E-cadherin, and promoted the acquisition of mesenchymal features, such as actin-rich protrusions (Figure 2). The opposite approach with ectopic expression of p115RhoGEF showed enhanced junctional E-cadherin and inhibition of protrusions (Figure 3). Our results suggest that p115RhoGEF contributes to the regulation of epithelial-mesenchymal balance in epithelial cells. The p115RhoGEF-dependent regulation of epithelial-mesenchymal balance appears to act on some, but not all of the determinants of epithelial plasticity. For example, promotion of mesenchymal features by p115RhoGEF depletion (enhanced actin-based protrusions in MCF7 cells) or inhibition of mesenchymal features by p115RhoGEF overexpression (reduced protrusion in MDA-MB-231) did not correspond with changes in the localization or expression of mesenchymal marker, vimentin (Figure S2A and S2B). In addition, changes in p115RhoGEF expression selectively altered the localization of E-cadherin at adherens junctions but had no effect on tight junctions (Figure S1). A gene expression study designed to identify factors responsible for epithelial-to-mesenchymal transition (EMT) found 55 genes that consistently displayed significant changes in expression across multiple EMT model systems [14]. p115RhoGEF was one of the 34 genes that were consistently downregulated as cells acquired a mesenchymal phenotype. Thus, our results indicate that changes in the expression of p115RhoGEF alone are not sufficient to induce/block EMT, it is likely that p115RhoGEF contribute to EMT in concert with other signaling pathways.

p115RhoGEF has been shown to specifically activate Rho proteins, and not Rac or Cdc42 [17], [18]. Experiments using FRET-based RhoA activity biosensor showed that p115RhoGEF overexpression activated RhoA in both MCF7 and MDA-MB-231, and the effects of p115RhoGEF were dependent on its GEF catalytic activity as expression of the catalytically inactive mutant p115RhoGEF (p115RhoGEF^DH mutant^) in MCF7 and MDA-MB-231 cells had no effect on epithelial plasticity, i.e. no change in adherens junctions or protrusions (Figure 5). However, p115RhoGEF can also promote nucleotide exchange on other Rho isoforms, such as RhoB and RhoC, and therefore is possible that the effects of p115RhoGEF are also mediated by multiple Rho isoforms. For example, Vega *et al* used siRNA to deplete cells of either RhoA or RhoC [5]. They found that depletion of the different Rho isoforms produced distinct changes to cell morphology and migration. RhoA-depleted cells were highly elongated with multiple Rac-dependent protrusions, while RhoC-depletion reduced cell migration and invasion by promoting the formation of broad lamellipodia. Their model proposed that RhoC activation, through the Rho effector FMNL3, promoted directional cell migration by restricting lamellipodial broadening; while RhoA regulated polarized migration by restricting excess Rac1 activity at the leading edge, an effect that was dependent on the Rho effectors, ROCK2. Identifying if there is differential activation of a particular Rho isoforms by p115RhoGEF could provide insight how p115RhoGEF regulates multiple aspects of epithelial plasticity.

Since Rho can activate multiple downstream effectors, we wanted to identify the effector(s) responsible for epithelial plasticity regulated by p115RhoGEF signaling pathway. Using a siRNA screen, we identified members of the DRF family of proteins (*DIAPH1*, *DIAPH2*, *DIAPH3* and *DAAM*) to be required for regulation of epithelial plasticity by p115RhoGEF (Figure 6). Earlier work using a similar siRNA screen of all known downstream RhoA effectors identified PRK2, but not DRF family members, as a regulator of epithelial junctional integrity [27]. One explanation for the discrepancy may be that the early screen used localization of occludin and ZO-1, both components of tight junctions, to identify changes in apical junction formation following effector depletion. We did not detect changes in ZO-1 localization at tight junctions following changes in p115RhoGEF expression in our system (Figure S1). This suggests that unique effectors downstream of Rho activation are utilized to regulate different components of epithelial morphology with one set regulating tight junction formation (ex. PRK2), while another family (ex. DRF family) is utilized to regulate adherens junctions and/or the actin cytoskeleton.

At this time, the data are not able to distinguish whether p115RhoGEF-dependent regulation of adherens junctions and protrusiveness are interconnected or independent events. The role of the DRF family in regulation of the actin polymerization is well known and our results are consistent with previous studies which have shown that mDia1-driven actin polymerization stabilizes adherens junctions and that mDia1 can regulate localization of adherens junction components such as E-cadherin in epithelial cells [21], [33], [34]. These previous reports, taken together with our data, support a model where p115RhoGEF-mediated changes in E-cadherin localization at adherens junctions are a consequence of DRF-mediated regulation of actin organization. However, at this time we cannot rule out that the effect of p115RhoGEF expressions on adherens junctions and protrusions are a result of two unique signaling events that occur downstream of the DRF family members. An additional layer of complexity is added when one considers that multiple RhoGEFs, activated by distinct signaling events, could converge to activate DIAPH1 at the cytoskeleton and/or cell-cell junctions. Cell-cell junctions are a critical feature of epithelial architecture, and it is likely that multiple RhoGEFs are involved in regulation of junctional integrity. For example, the RhoGEFs, p114RhoGEF and Ect2, have both been shown to regulate epithelial junctional integrity through activation of the Rho effector, ROCK2. Thus, activation of a common Rho effector, like DIAPH1, by p115RhoGEF and other RhoGEFs could impart differential regulation of junctional integrity by coupling each RhoGEF to specific upstream stimuli. Future experiments will help to identify the exact signaling cascades responsible for the observed effects of p115RhoGEF expression junctional integrity.

Studies in drosophila have also shown RhoGEF2, which is the homologous RGS RhoGEF to signal through Dia to regulate E-cadherin endocytosis [35]. Furthermore, loss of either Dia or RhoGEF2 have been shown to disrupt junctions and enhance protrusions during embryonic development providing genetic evidence to support a model in which RhoGEF2 and Dia could be a part of the same pathway [36]. Our results suggest that since DRFs are required for p115RhoGEF-mediated regulation of adherens junctions and protrusions, p115RhoGEF could be the human homolog of RhoGEF2 that regulates epithelial plasticity in drosophila.

In conclusion, we present evidence that p115RhoGEF regulates epithelial plasticity by regulation of adherens junctions and protrusions. p115RhoGEF activates RhoA and requires members of the DRF family to regulate epithelial plasticity. Other RhoGEFs such as p114RhoGEF, Ect2, GEF-H1, and PRG have also been shown to regulate either junctional integrity or membrane protrusions in epithelial-like cells [15], [24], [37], [38]. Thus, multiple RhoGEFs may contribute towards regulation of epithelial plasticity where each RhoGEF could be linked to a specific upstream stimuli to regulate a distinct Rho signaling module [9]. Our data has identified a novel role for p115RhoGEF as a potential regulator of Rho-DRF signaling module that regulates epithelial plasticity. Future research could be directed towards integrating these individual Rho modules into comprehensive signaling circuits that control a complex process such as epithelial plasticity.

## Materials and Methods

### Reagents and Antibodies

Collagen-1 was purchased from either BD Biosciences (Bedford, MA) or Invitrogen (Carlsbad,CA). E-cadherin antibody was obtained from BD Biosciences. p115RhoGEF, LARG and PRG antibodies were purchased from Santa Cruz (Santa Cruz, CA). Antibody for β-actin was purchased from Cell Signaling (Danvers, MA). Monoclonal anti-vimentin antibody (clone V9) was purchased from Sigma-Aldrich, ZO-1 antibody was purchased from Invitrogen (Carlsbad,CA), hDia1(human) antibody was purchased from Proteintech (Chicago, IL). Lipofectamine 2000 and RNAiMAX was purchased from Invitrogen. siRNA oligonucleotides (p115RhoGEF: CATACCATCTCTACCGACG, LARG: GAAACTCGTCGCATCTTCC and PRG: ACTGAAGTCTCGGCCAGCT) [41] were synthesized by Dharmacon. ON-TARGETplus Non-targeting siRNA was purchased from Dharmacon. siGENOME SMARTpool siRNAs library to target RhoA effector genes [27] and individual siRNAs to target *DIAPH1*: *DIAPH1* oligo#1 GAAGUGAACUGAUGCGUUU and oligo #2 GAUAUGAGAGUGCAACUAA were purchased from Dharmacon. pQCXIB-CMV/TO-DEST (w320) created by Dr Eric Campeau and pBabe-puro-Rho biosensor (created by Klaus Hahn) were obtained from Addgene. myc-DEST was a gift from Dr Adi Dubash.

### Cell Culture

MCF7, MCF10A and MDA-MB-231 cells were obtained from ATCC (Manassas, VA). MCF7 breast carcinoma cells were routinely cultured in DMEM supplemented with 10% FBS, glutamine, insulin, non-essential amino acids and antibiotics. MCF10A cells were cultured in DMEM/F12 supplemented with 5% horse serum, 20 ng/ml EGF, 0.5ug/ml Hydrocortisone, 100ng/ml Cholera Toxin, insulin 10ug/ml and antibiotics. Assay media used for MCF10A migration assay contained DMEM/F12 supplemented with 2% horse serum, 0.5ug/ml hydrocortisone,100 ng/ml cholera toxin, 10ug/ml insulin and antibiotics. MDA-MB-231 cells were obtained from ATCC (Manassas,VA) and Phoenix ectopic retrovirus packaging cell line (created by Gary Nolan Lab obtained from National Gene Vector Biorepository) were cultured in DMEM supplemented with 10% FBS and antibiotics.

### Generation of p115RhoGEF stable cell lines

myc-tagged p115RhoGEF was PCR amplified and cloned in retroviral vector pQCXIB- CMV/TO-DEST (w320) using Gateway cloning protocol. The resulting pQCXIB-myc-p115RhoGEF was transfected into Phoenix cells ectropic packaging cell line using calcium phosphate. Fresh media was added 16 hrs after transfection and virus was collected at 36 hrs and 48 hrs after transduction. The supernatant was filtered with a 0.45um filter and either used fresh or stored at −80°C. MCF7 cells were infected with viral supernatant plus 6ug/ml polybrene. Next day media was replaced and cells were selected with 8ug/ml blasticidin to select for cell population that expressed myc-p115RhoGEF. To generate the siRNA resistant variant of p115RhoGEF, we made three silent point mutations in the region targeted by the siRNA which rendered it resistant to knockdown and cloned it in pQCXIB-CMV/TO-DEST(w320) and used retroviral transduction to make MCF7 cells that stably expressed this construct. To make the catalytically inactive mutant form of p115RhoGEF (p115RhoGEF^DHmutant^), we made a Y568F mutation in wild type p115RhoGEF and cloned it in pQCXIB-CMV/TO-DEST(w320). We used retroviral transduction to generate a cell line that stably expressed this construct by selection for resistance to blasticidin.

### Immunoblotting and Immunofluorescence

Immunoblots were performed as previously described [39] and images analyzed using Bio-Rad Quantity One software and Adobe Photoshop. Cells were fixed and stained as previously described [40].

Laser confocal images were acquired with an Olympus BX51 Laser scanning confocal microscope outfitted with their Fluoview1000 system. Images were visualized through a 60× oil immersion objective. Images were acquired and processed with Fluoview1000 software and exported as BMP files for further editing with Adobe Photoshop. Junctional staining intensity for experiments involving p115RhoGEF^DH mutant^ was calculated by drawing a line along cell-cell junctions and the integral staining intensity along this line was measured using Fluoview software.

E-cadherin ratio (Ratio of junctional over cytoplasmic E-cadherin) for Figures 2, 3 and 6 was calculated by dividing junctional E-cadherin staining intensity by cytoplasmic E-cadherin staining intensity. Cytoplasmic staining intensity was measured by drawing a line along the inner edge of junctions. E-cadherin intensity of the entire cell was calculated by drawing a line along the outer edge of the junction. Junctional E-cadherin was measured by subtracting cytoplasmic E-cadherin intensity from E-cadherin intensity of the entire cell. Junctional E-cadherin intensity for each cell was divided by 2 to account for the contribution of both the cells forming the junction. E-cadherin ratio was then obtained by dividing junctional E-cadherin staining intensity by cytoplasmic E-cadherin staining intensity. E-cadherin ratio was plotted as mean ± S.E. p-value was calculated using 2 tailed students t-test. Junctional staining intensity for Figure 5 was obtained by drawing a line along the length of the cell-cell junction using Fluoview 1000 software and calculating the staining intensity of E-cadherin per µm of junctional length. E-cadherin junctional staining intensity was plotted as mean ± S.E. and p-value was calculated using 2 tailed students t-test.

### FRET imaging of RhoA activity

MCF7 and MDA-MB-231 cells were transfected with the RhoA biosensor probe (pBabe-puro-Rho biosensor). 18 hrs post-transfection, cells were imaged in phenol red-free DMEM. Confocal images were acquired with an Olympus BX51 Laser scanning confocal microscope outfitted with their Fluoview1000 system. Images were visualized through a 60× oil immersion objective. A 405 nm Ar/HeNe laser refined by a 405/473 dichroic mirror was used excite CFP, and the CFP and FRET (CFP) emission signals were simultaneously recorded using BA465-495 nm (CFP) and BA535-565 nm (FRET) emission filters. The FRET emission image was divided by the CFP image to create a ratiometric image, which is reflective of the RhoA activity within the cell. A linear pseudocolor lookup table was applied to all ratio images with warm and cool colors representing high and low RhoA activity, respectively. Ratio values were normalized to the lower scale value, which was chosen to exclude the bottom 7% of the total histogram distribution to avoid areas of low signal-to-noise ratio [19], [20]. Images were acquired and processed with Fluoview 1000 software, and exported as BMP files for further editing with Adobe Photoshop. Total RhoA activity was quantitated by calculating FRET intensity per µm^2^ of the area of cell. Junctional RhoA activity was quantitated by calculating FRET intensity per µm of junction length.

### Cell Migration

Cell migration in MCF7 and MCF10A was measured using a scratch wound assay. Cells were transfected with p115RhoGEF siRNA using RNAiMAX according to manufacturer's instructions. To measure effect of overexpression of p115RhoGEF on cell migration, stable overexpressors of p115RhoGEF were used. Migration assays were begun 72 hrs after siRNA transfection. To start the assay, monolayers were scratched with a 10 µl micropipette tip to form a wound and culture media was replaced with complete media plus 1 µg/ml mitomycin for MCF7 cells and assay media for MCF10A cells. Initial (t = 0 hrs) phase contrast images were taken after which cells were maintained at 37°C, 5% CO2. Final phase contrast images were taken at t = 24 hrs. Images were captured using an Olympus IX71 microscope with a 10× objective. For single cell migration assay, MDA-MB-231 cells were plated on collagen-coated MatTek dishes and maintained at 37°C, 5% CO2 using live cell environmental chamber (NEUE Group, Ontario, NY). Cell position of 5–6 cells per field was determined using Slidebook software and x-y co-ordinates of individual cells were plotted with starting points adjusted to (0,0). Total displacement was calculated using Slidebook software.

## Supporting Information

Figure S1 **Changes in the expression levels of p115RhoGEF does not affect tight junctions in MCF7 cells.** A) p115RhoGEF was depleted in MCF7 and immunofluorescence was used to detect the tight junction marker, ZO-1, and adherens junction marker, E-cadherin. ZO-1 localization and intensity was similar in both control and p115RhoGEF-depleted MCF7 cells. This is in contrast to changes in distribution of E-cadherin in p115RhoGEF-depleted MCF7 cells. Scale bars  = 10 µm B) p115RhoGEF overexpressed in MCF7 cells and immunofluorescence was used to detect the tight junction marker, ZO-1, or the adherens junction marker, E-cadherin. ZO-1 localization and intensity was similar in both control and p115RhoGEF-OE MCF7 cells. No change in the localization and intensity pattern of ZO-1 was observed, which is in contrast to the enhanced junctional localization of E-cadherin upon overexpression of p115RhoGEF. Scale bars  = 20 µm.
(TIF)

Figure S2 **Change in expression of p115RhoGEF does not lead to change in expression of vimentin in MCF7 and MDA-MD-231 cells.** A) Knockdown or overexpression of p115RhoGEF in MCF7 and MDA-MB-231 cells did change in the localization and intensity of vimentin localization. Scale bars  = 10 µm. B) Immunoblot for vimentin in p115RhoGEF-depleted MCF7 cells, or MDA-MB-231 cells that overexpress p115RhoGEF did not show any change in the expression levels of vimentin.
(TIF)
