# Supplementary figures and images for: A Novel Role for p115RhoGEF in Regulation of Epithelial Plasticity
Source: PLoS One. 2014 Jan 23;9(1):e85409. doi: 10.1371/journal.pone.0085409 (PMC3900421; doi:10.1371/journal.pone.0085409)

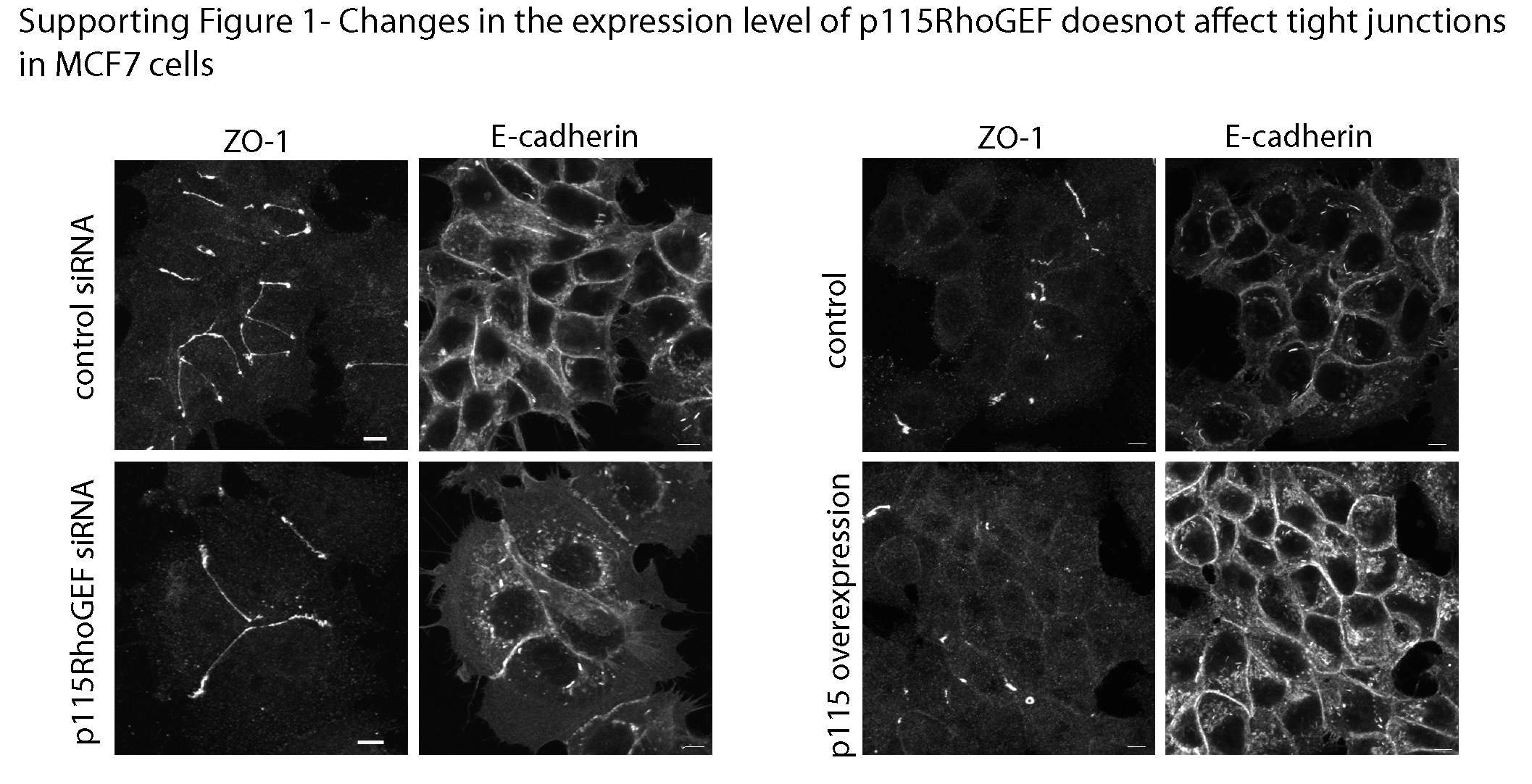

Supplement: Figure S1 — Changes in the expression levels of p115RhoGEF does not affect tight junctions in MCF7 cells. A) p115RhoGEF was depleted in MCF7 and immunofluorescence was used to detect the tight junction marker, ZO-1, and adherens junction marker, E-cadherin. ZO-1 localization and intensity was similar in both control and p115RhoGEF-depleted MCF7 cells. This is in contrast to changes in distribution of E-cadherin in p115RhoGEF-depleted MCF7 cells. Scale bars = 10 µm B) p115RhoGEF overexpressed in MCF7 cells and immunofluorescence was used to detect the tight junction marker, ZO-1, or the adherens junction marker, E-cadherin. ZO-1 localization and intensity was similar in both control and p115RhoGEF-OE MCF7 cells. No change in the localization and intensity pattern of ZO-1 was observed, which is in contrast to the enhanced junctional localization of E-cadherin upon overexpression of p115RhoGEF. Scale bars = 20 µm. (TIF) [file pone.0085409.s001.tif]

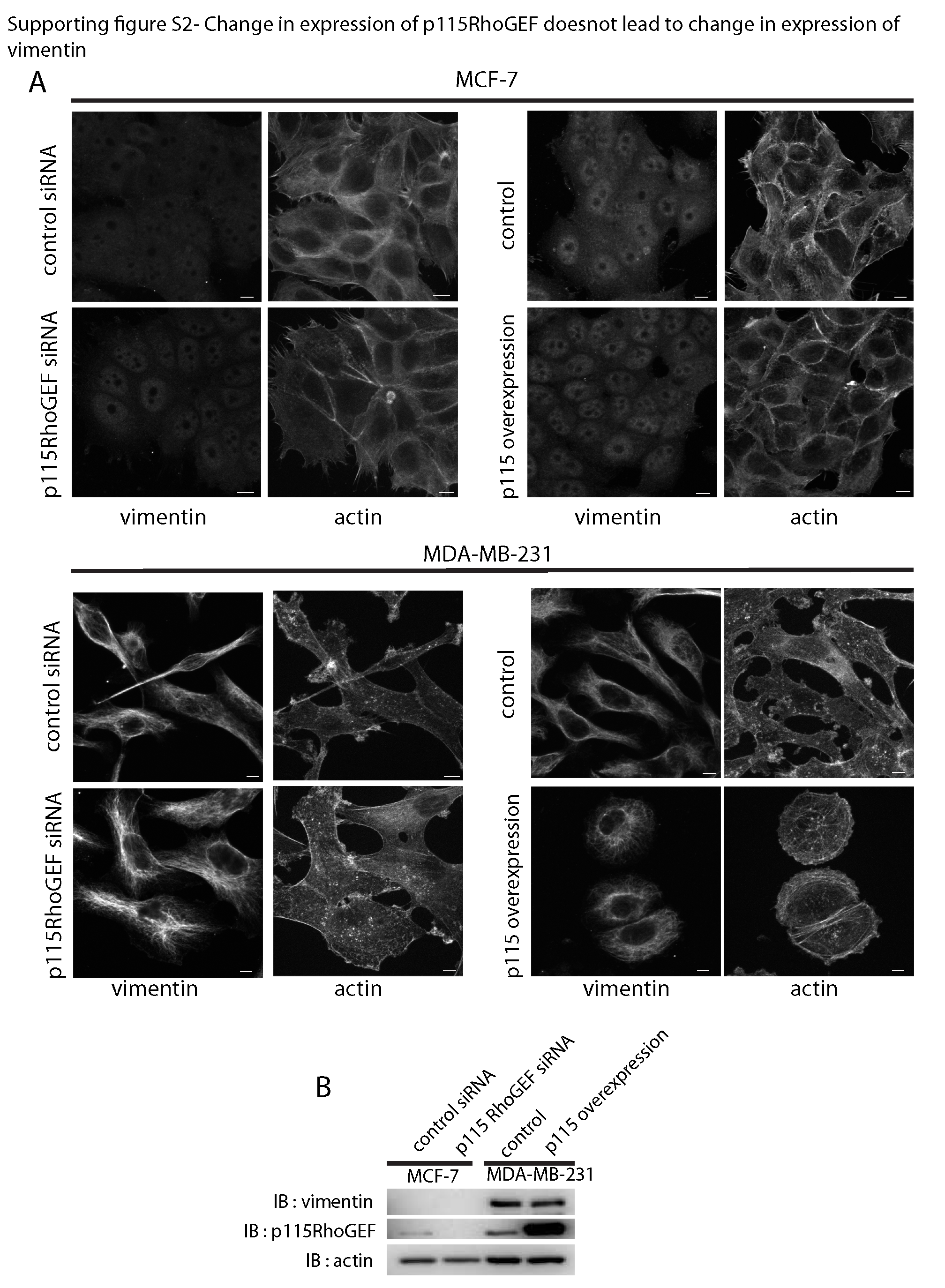

Supplement: Figure S2 — Change in expression of p115RhoGEF does not lead to change in expression of vimentin in MCF7 and MDA-MD-231 cells. A) Knockdown or overexpression of p115RhoGEF in MCF7 and MDA-MB-231 cells did change in the localization and intensity of vimentin localization. Scale bars = 10 µm. B) Immunoblot for vimentin in p115RhoGEF-depleted MCF7 cells, or MDA-MB-231 cells that overexpress p115RhoGEF did not show any change in the expression levels of vimentin. (TIF) [file pone.0085409.s002.tif]
